# Utility of Inflammatory Markers in Predicting Hepatocellular Carcinoma Survival after Liver Transplantation

**DOI:** 10.1155/2019/7284040

**Published:** 2019-10-14

**Authors:** Media N. Ismael, Justin Forde, Eduardo Milla, Walid Khan, Roniel Cabrera

**Affiliations:** ^1^Department of Medicine, Internal Medicine Residency, University of Florida, Gainesville, FL, USA; ^2^Department of Medicine, Division of Gastroenterology, Hepatology and Nutrition, University of Florida, Gainesville, FL, USA

## Abstract

Inflammatory markers have been studied in cancers and chronic states of inflammation. They are thought to correlate with tumor pathology through disruption of normal homeostasis. Markers such as neutrophil to lymphocyte ratio (NLR) among others have shown promise as prognostic tools in various cancers. In this study, we evaluate complete blood count based inflammatory markers in hepatocellular carcinoma (HCC) to predict overall and recurrence-free survival of patients after liver transplant. Between 2001 and 2017, all HCC indicated liver transplants were retrospectively reviewed. Inclusion criteria included presence of complete blood cell counts with differential within three months prior to transplantation. Exclusion criteria included retransplantation and inadequate posttransplant followup. A total of 160 patients with HCC were included in the study. Of those, 74.4% had hepatitis C virus as the underlying cause of HCC. Calculated Model for End stage Liver Disease (MELD) scores were statistically worse in patients with elevated NLR (≥5), derived NLR (≥3), and low lymphocyte to monocyte ratio (LMR) (<3.45), whereas elevated platelet to lymphocyte ratio (PLR) (≥150) did not correlate with MELD. Of the tumor characteristics, low LMR was associated with tumor presence and microvascular invasion on explant. Though overall survival trended towards better outcomes with low NLR and dNLR and high LMR, these did not reach statistical significance. High LMR also trended towards better recurrence-free survival without statistical significance. Low PLR was associated with statistically significant overall and recurrence-free survival. In conclusion, while prior studies in HCC have identified NLR as surrogate for tumor burden and survival, in this study we highlight that PLR is a good surrogate of mortality and recurrence-free survival in HCC transplant patients. Further, future study of PLR, NLR, and LMR in larger HCC populations before and after interventions may help clarify their clinical utility as a simple and noninvasive clinical tool as prognostic markers.

## 1. Introduction 

Hepatocellular carcinoma (HCC) is the fourth leading cancer in mortality and the sixth most common cancer worldwide [1]. According to the Scientific Registry of Transplant Recipients, there is a steady growth in liver transplant (LT) numbers with an annual 3% increase as of 2017. Of those on the wait list for LT, HCC is on the rise as an indication and accounts for nearly 10% of transplants in the same report [2]. HCC recurrence risk is perpetuated by the strong association with cirrhosis and the high vascular nature. This limits long term outcomes of localized treatment options such as resection and ablative therapy, and thus LT remains the cornerstone curative treatment in management of HCC [3, 4]. Despite the growth in LT numbers, donor supply is a restrictive factor and there is continued need to identify HCC patients who derive the major benefit from LT.

Transplanted patients who suffer from HCC recurrence have a rough median survival of about a year after recurrence. One approach to mitigate this risk was the development of the Milan criteria, with which the risk of recurrence after LT is estimated to be around 10-20% [3, 5–8]. In recent years, the use of the Milan criteria has been shown to be unreliable in predicting recurrence after LT [9, 10]. In contrast, tumor size, response to local ablative treatment, alpha-fetoprotein (AFP), C-reactive protein, and microvascular invasion may better correlate with recurrence [7, 11–13]. Nevertheless, most of these factors still fall short due to their collection in the posttransplant period and need for explant pathology.

Multiple studies have attempted to identify pretransplant predictors of HCC recurrence to improve patient selection and transplant outcomes. The development of cancer leads to neoangiogenesis and sets the stage for chronic inflammatory response that disrupts the normal immunological pathway [14]. Involvement of leukocytes and platelets, along with recruitment of interleukins and growth factors, mediates tumor growth [9, 13]. This disruption in normal homeostasis in these pathways can be detected with simple, easy to measure markers that may serve as surrogates of cancer aggressiveness and survival. The use of inflammatory marker ratios obtained from complete blood count (CBC) testing has been applied in different cancers and chronic states of inflammation [15–17]. We and others have previously published that the neutrophil to lymphocyte ratio (NLR) can be utilized to predict HCC recurrence [18]. In this study, we assess the ability of NLR as well as other CBC derived inflammatory markers to predict overall and recurrence-free survival in all comers with HCC undergoing liver transplantation over a 17-year period.

## 2. Materials and Methods

### 2.1. Study Population

Liver transplant registry at the University of Florida in Gainesville, Florida, was used to identify adult patients who underwent LT with indication of HCC during the period of 2001-2017. The study was approved by the hospital institutional review board. In a retrospective review of the institute's electronic medical records, patient locoregional therapy prior to transplant, demographics at the time of transplant, tumor pathology, and posttransplant survival were obtained. Inclusion criteria included presence of CBC with differential within three months prior to transplantation and tumor burden within Milan criteria at time of transplant. Exclusion criteria included retransplantation, subsequent diagnoses of non-HCC malignancy (primarily cholangiocarcinoma) on explant pathology, and inadequate posttransplant followup.

### 2.2. Inflammatory Surrogate Criteria

Routine CBC prior to transplant was used to obtain white cell count differential and platelets. The relative increases in neutrophils, monocytes, and platelets, along with decrease in lymphocytes in the inflammatory beds, were used as surrogate markers for inflammation and were calculated as neutrophil to lymphocyte ratio (NLR), derived neutrophil to lymphocyte ratio (dNLR) utilizing absolute neutrophil count for the derivation, lymphocyte to monocyte ratio (LMR), and platelet to lymphocyte ratio (PLR). Each surrogate marker was divided into two groups for comparison. The cutoff values were based on prior publications, including NLR of 5 [18], dNLR of 3 [15], LMR of 3.45 [19], and PLR of 150 [20].

### 2.3. Tumor Characteristics, Followup after Transplant, Tumor Recurrence, and Survival

AFP tumor marker for all patients was collected prior to transplant. These data were included if they were obtained within 6 months of transplant date. Pretransplant treatments including locoregional therapy and/or resection for all patients were reviewed. As the study period spanned across different MELD scoring patterns, to unify the data, we collected the set of creatinine, total bilirubin, INR, and sodium values for each patient to calculate MELD scores at transplant.

Following transplant, all explanted livers were evaluated and discussed in a multidisciplinary board review including hepatologists and pathologist. These reviews were studied and data including evidence of tumor cells on explant, histopathological grade and stage, and microvascular invasion was collected. If no tumor was found due to ablative treatment/necrosis, this was marked as no tumor on explant. If histopathological grade was not assessed due to lack of enough tissue pathology, we marked those grades as no histopathology on explant despite evidence of tumor cells. This can explain discrepancy between evidence of tumor on explant and total patients with evidence of histological grade.

After transplant, patients were followed in clinic and monitored by a hepatologist at least every three months in the first year, followed by every six months for the next two years and yearly thereafter. These visits allowed detection of biochemical and clinical changes in patient status such as evidence of LFT abnormalities. HCC recurrence screening protocol included AFP measurement and imaging every six months for the first three years after transplant. Patients were followed for five years after transplant or until end of study time or death whichever occurred first.

### 2.4. HCC versus Non-HCC Cirrhosis and Relation to Inflammatory Surrogate Markers

In order to validate the correlation of inflammatory surrogates to HCC versus underlying cirrhosis, we performed a subset analysis to compare the role of these surrogates in survival of non-HCC transplant by using a 1:1 Propensity matching to our HCC patients based on age and gender.

### 2.5. End Points and Statistical Analysis

Primary outcomes of study were effect of inflammatory surrogates on five-year overall and recurrence-free survivals. Secondary endpoint was correlation of biomarkers to established prognostication factors such as AFP, MELD scores, and tumor pathology.

SPSS version 25.0 was used for statistical analysis. The *χ*2 test was used to compare categorical variables and t-test was used to compare continuous variables. Overall survival (OS) was defined from time of transplant to death or end of study. Recurrence was diagnosed based on imaging or biopsy proven recurrence of cancer. Recurrence-free survival (RFS) was defined from time of transplant to recurrence or censored for recurrence at end of study or death. End of study was defined as 5 years from transplant.

Univariate and multivariate analysis to estimate hazard ratio were calculated using Cox regression analysis. Factors were included in multivariate analysis if P value was <0.1 in the univariate analysis. Kaplan Meier was used to estimate overall and recurrence-free survival.

## 3. Results

### 3.1. Patient Characteristics

Over the study timeframe, 212 LT were performed at our center for HCC. After applying inclusion and exclusion criteria, 160 transplants were included in the study. Main reasons for being excluded were lack of CBC within 3 months prior to transplant and/or lack of appropriate followup after transplant. For the 160 patients, median age at LT was 58 years, median MELD 11.3, with 78.1% of these patients being male, 76.9% Caucasian, and 74.4% with HCV as the underlying etiology. Of these 160 patients, only 30 (18.8%) did not receive a form of locoregional therapy prior to transplant.  Table 1 details the characteristics of each inflammatory surrogate subgroup. Only MELD scores were noted to be different based on the inflammatory surrogate markers.

### 3.2. Tumor Pathology

Overall, 80.6% of patients had viable tumor present on explant. In retrospective assessment of tumor histology, only two patients had poorly differentiated tumor; therefore we combined those two with moderate differentiated pathology in one group for analysis. Moderate or poorly differentiated tumor occurred in 47.5%, and well differentiated tumor in 22.5%. T2 tumor pathology was most dominant on explant accounting for 46.9%, followed by T1 at 36.3% and then T3 with 3.8%. Patients with AFP greater than 300 accounted for 6.9% of LT. Microvascular invasion was noted in 15.6% of all patients. Tumor characteristics and correlation to inflammatory surrogates are evaluated in Table 2. Low LMR was noted to significantly correlate with tumor pathology and high PLR trended towards worse tumor pathology.

### 3.3. Overall Survival and Mortality

**Figure 1:**
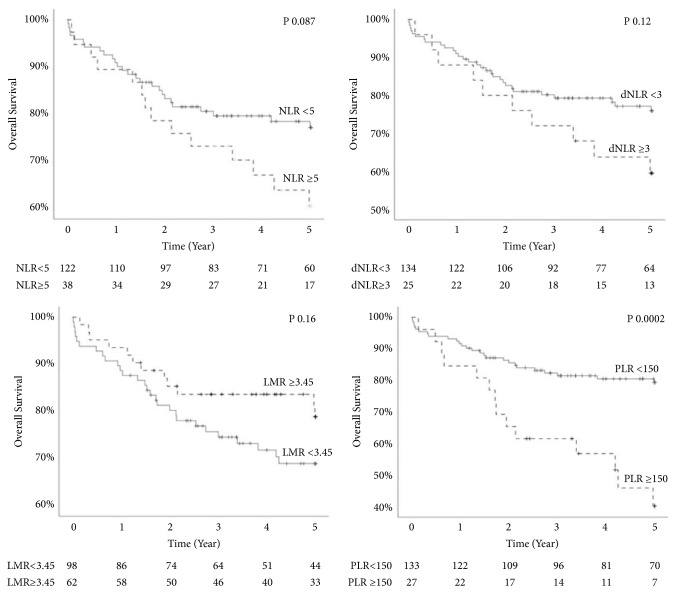
Effect on inflammatory markers on 5-year overall survival.

**Figure 2:**
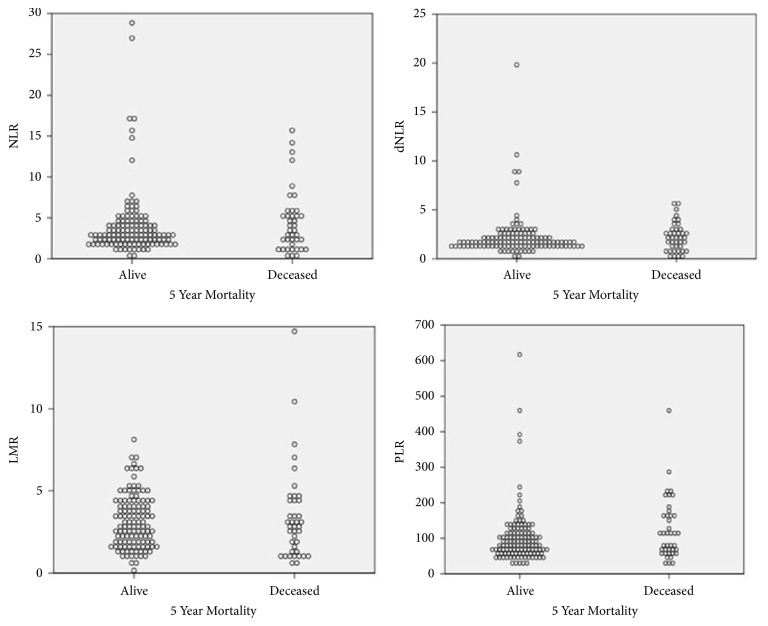
Inflammatory biomarkers dispersion and overall survival.

At the end of study period, 40 patients (25%) passed away with median survival of 4.97 years and a 5-year OS rate of 72.9%. The major causes of death included sepsis and end organ damage followed by HCC recurrence. Univariate analysis of patient and tumor characteristics effect on OS are detailed in Table 3. Factors significantly correlating with poor OS included AFP >300 and microvascular invasion on tumor explant. Of the inflammatory markers, higher NLR, dNLR, and PLR and lower LMR show worse OS and higher hazard ratio but of these, only PLR was statistically significant. Multivariate analysis of NLR, PLR, AFP, and microvascular invasion showed elevated hazard ratio for all included criteria with P values of 0.9, 0.01, 0.33, and 0.09 respectively. Kaplan Meier's 5-year OS stratified by inflammatory surrogates are shown in Figure 1. Dispersion of inflammatory surrogate markers and survival is shown in Figure 2.

### 3.4. Recurrence and Recurrence-Free Survival

**Figure 3:**
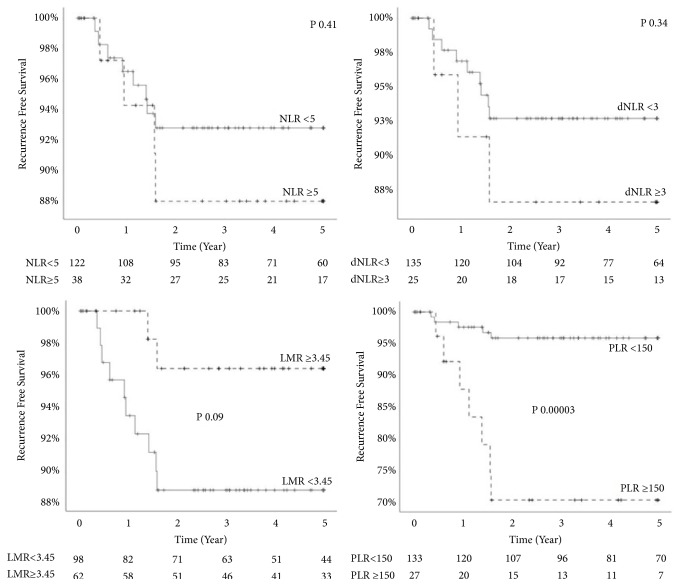
Effect on inflammatory markers on 5-year recurrence-free survival.

**Figure 4:**
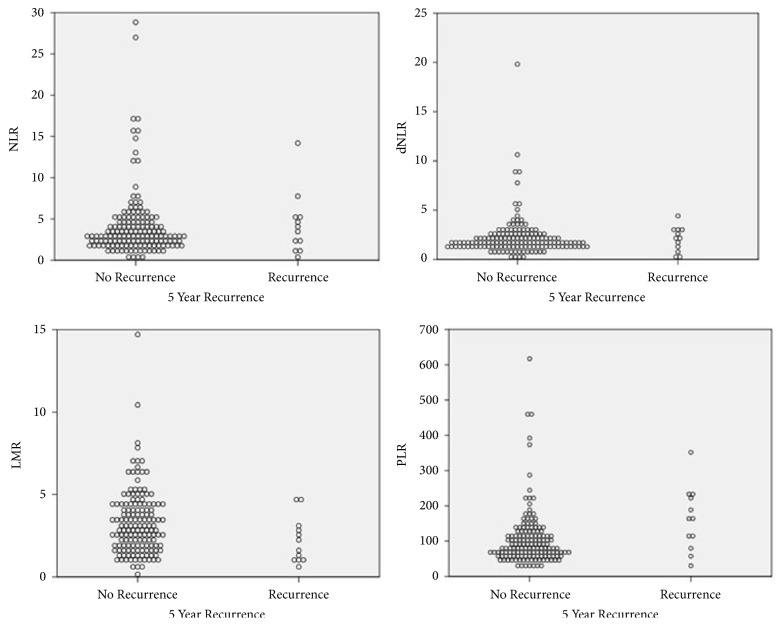
Inflammatory markers dispersion and recurrence-free survival.

Twelve patients (7.5%) had recurrence by the end of study period. All recurrences occurred within the first two years after LT. Median recurrence-free time was 4.87 years and 5-year RFS rate was 91.7%. Seven of the recurrences occurred as extrahepatic metastasis and the rest recurred in the transplanted liver. Univariate analysis of patient and tumor characteristics effect on RFS are detailed in Table 3. Factors significantly correlating with poor RFS included race other than white, AFP >300, and microvascular invasion on tumor explant. Of the inflammatory markers, higher NLR, dNLR, and PLR and lower LMR show worse RFS and higher hazard ratio but of these, only PLR was statistically significant. Multivariate analysis of PLR, race, AFP, and microvascular invasion showed elevated hazard ratio for all included criteria with P values of 0.001, 0.005, 0.15, and 0.07 respectively. Kaplan Meier's 5-year RFS stratified by inflammatory surrogates are shown in Figure 3. Dispersion of inflammatory surrogate markers and recurrence is shown in Figure 4.

### 3.5. Non-HCC Cirrhosis and Inflammatory Markers

Using the 1:1 Identified cases of non-HCC transplanted patients, we noted an overall 5-year survival of 89.8% after transplant in non-HCC patients. Only LMR of the surrogate markers correlated with 5-year survival (88.4% vs 90.7%, P 0.58 for NLR≥5; 92.3% vs 88.9%, P 0.51 for dNLR≥3; 97.8% vs 87.1%, P 0.04 for LMR≥3.45; and 87.8% vs 90.5%, P 0.59 for PLR≥150).

## 4. Discussion

HCC is a highly angiogenic tumor that arises in the setting of chronic inflammation and cirrhosis. The role of inflammation in cancer development has been under research for decades and recent focus has been on attempts to utilize surrogate markers of inflammation to predict tumor virulence and survival. The NLR and other peripheral marker ratios calculated from white cell count differential could serve as systemic inflammatory surrogates for tumor biology, aggressiveness, and risk for adverse outcomes. In this study, we examine long-term outcomes of patients with HCC undergoing liver transplant regardless of etiology and its relationship to various inflammatory surrogates derived from ratios of the differential white blood cell count. While we note that the NLR and dNLR are not reliable predictors of long-term HCC outcome (OS and RFS) at 5-years, we did find a correlation between LMR and microvascular invasion. Further, our study shows PLR to be an independent predictor of long-term survival in patients receiving a liver transplant for HCC.

Neutrophils along with monocytes and platelets play important role in inflammatory responses to injury, infection, or tumor. Their activation and migration to damaged tissue lead to tissues growth and angiogenesis in attempts from the immune system to repair wound. Lymphocytes act as regulatory cells in inflammatory states, activating prohealing cytokines. An imbalance between these cells specifically suppression of lymphocytes and increased activation of platelets, neutrophils, and monocytes can promote ongoing injury and tumor growth [14, 21]. In a number of studies, the NLR has been shown to correlate with survival in solid tumors [17, 18]. Our group and others have previously shown elevated NLR to correlate with OS and RFS in HCC after LT [18, 22, 23]. In instances when NLR can't be derived due to a missing lymphocyte count, calculating the dNLR may be a useful alternative [24]. In our study, patients with both NLR ≥5 and dNLR ≥3 showed a higher rate of microvascular invasion and worse long-term outcomes; however these were not clinically significant (5-year OS: 60.5% vs 77.2%; and RFS: 88% vs 92.8%). Other studies such as Parisi et al.'s have also noted a lack of significant correlation between NLR and survival [10]. The lack of standardized timing (up to three months prior to LT) of when to obtain CBC may contribute to the inconsistent observations. In addition, the severity of underlying cirrhosis and compromised liver function by HCC burden could be another factor to explain the elevations in NLR and dNLR in our population [25]. The finding supporting this explanation is shown by the correlation between patient's MELD score and the NLR/dNLR with a statistical trend of worse OS (P 0.09 and 0.12). In our subgroup analysis, non-HCC patients with underlying cirrhosis did not have a correlation between NLR/dNLR and survival. Further studies to include MELD scores for all transplanted patients as well as a larger study size may help better delineate these correlations.

The LMR is another useful marker that has been investigated as an indicator of survival in solid tumors [19, 26, 27]. While there is ambiguity regarding the molecular processes behind the impact of LMR, low lymphocyte and high monocyte counts are implicated in cytokine production aiding in tumor progression [28]. In addition, elevated monocyte counts are correlated with microvascular invasion and poorer prognosis of HCC [29]. These findings are boosted by our results showing patients with higher LMR (≥3.45) having significantly lower rates of tumor present in the explant with less microvascular invasion. Previous studies show a correlation between high LMR and improved OS and RFS in HCC after LT [26, 30, 31]. While we observed improved 5-year OS (78.8% vs 68.9%) and RFS (96.4% vs 88.7%) with higher LMR in our population, these findings did not reach significance. The severity of underlying liver disease likely contributes to this as we noted in our subgroup analysis of non-HCC patients. For example, Raffetti et al. note CBC derived markers are not predictive of cancer development in HIV patients who are in chronic inflammatory state [32]. Smaller sample size and the dynamic nature of CBC values preceding LT may explain some of the other differences in reaching statistical significance.

Platelet activation releases cytokines that aid with tumor angiogenesis. Platelets combined with lymphocytes as the PLR have been studied as an additional prognostic factor for survival in cancer [20, 33, 34]. Similar to prior publications, we found PLR ≥150 to be a strong predictor of worse 5-year OS (40.2% vs 79.4%) and RFS (70.2% vs 95.9%) in HCC patients with hazard ratio of 3.18 for OS and 7.95 for RFS [22, 35, 36]. In addition, elevated PLR correlated closely with AFP >300 (P 0.07) and tumor presence on explant (P 0.08) which are well-established predictors of survival [7, 11]. On multivariate analysis, PLR does continue to show significant correlation with OS and RFS. Interestingly, in contrast to other CBC derived inflammatory markers, PLR was not associated with underlying MELD score. Though the mechanism is unclear, these findings suggest PLR could better reflect cancer specific survival in tumors with background of underlying inflammation such as HCC and cirrhosis.

One remarkable incidental finding was evidence of worse RFS in patients who were of non-white races. This may correlate with patient's health literacy as well as financial means to followup in clinic and warrants further investigations.

In this study, most patients (130) underwent at least one form of locoregional therapy prior to transplant. Though this may be considered a confounding factor, we propose differently. Given the waitlist for transplant, different forms of ablative therapy are standard practice to bridge treatment and are in line with current guidelines [3, 4]. If we were to exclude these patients, the study results would not reflect real-life conditions.

Notable limitations to our research are the retrospective nature of the study and that it reflects a single center experience. In addition, choosing three months as the window for CBC may have resulted in some variability in results; however, a narrower time frame would have led to an underpowered analysis. A growing number of cancer studies show the importance of systemic inflammatory surrogate markers in the prognosis of patients. Our study is an important addition to the growing evidence that such markers may serve to prognosticate and predict the outcome of HCC patients after LT.

## 5. Conclusion

While prior studies in HCC have identified NLR as a tumor surrogate, in this study we highlight that PLR is a good surrogate of mortality and recurrence-free survival in HCC LT patients. This work supports further study of PLR in combination with other pretransplant markers, such as AFP and imaging, to determine if its addition into a model better captures transplant eligibility and benefits. Further, future study of PLR, NLR, and LMR in larger HCC populations before and after interventions may help clarify their clinical utility as a simple and noninvasive clinical tool as prognostic markers.

## Figures and Tables

**Table 1:** Patient characteristics and correlation with inflammatory markers.

	NLR			dNLR			LMR			PLR		
	<5	≥5	P	<3	≥3	P	<3.45	≥3.45	P	<150	≥150	P
N	122	38		135	25		98	62		133	27	
Male	78.7%	76.3%	0.76	78.5%	76%	0.78	78.6%	77.4%	0.86	78.9%	74.1%	0.58

Age (Year) mean ± SD	57±8	58±7	0.76	57±8	57±8	0.92	58±7	57±8	0.54	57±8	58±7	0.80

White race	74.6%	84.2%	0.22	75.6%	84%	0.36	78.6%	74.2%	0.52	76.7%	77.8%	0.90

HCV etiology	75.4%	71.1%	0.59	74.1%	76%	0.84	70.4%	80.6%	0.15	74.4%	74.1%	0.97

MELD, mean ± SD	12±7	17±10	**0.004**	12±7	18±11	**0.02**	16±8	9±6	**0.003**	13±8	12±8	0.57

dNLR, derived neutrophil lymphocyte ratio; HCV, hepatitis C virus; LMR, lymphocyte monocyte ratio; MELD, Model for End Stage Liver Disease; NLR, neutrophil lymphocyte ratio; PLR, platelet lymphocyte ratio.

**Table 2:** Tumor Characteristics and correlation with inflammatory markers.

		NLR			dNLR			LMR			PLR		
		<5	≥5	P	<3	≥3	P	<3.45	≥3.45	P	<150	≥150	P
N		122	38		135	25		98	62		133	27	
AFP >300		6.6%	7.9%	0.78	6.7%	8%	0.81	9.2%	3.2%	0.15	5.3%	14.8%	**0.07**

Tumor on explant		78.7%	86.8%	0.27	80.7%	80%	0.93	85.7%	72.6%	**0.04**	78.2%	92.6%	**0.08**

Histopathologic Stage (%)	*T1*	37.7%	31.6%	0.85	37.8%	28%	0.82	35.7%	37.1%	0.37	36.8%	33.3%	0.53
	*T2*	45.1%	52.6%		45.9%	52%		49%	43.5%		45.9%	51.9%	
	*T3*	4.1%	2.6%		3.7%	4%		5.1%	1.6%		3%	7.4%	

Histologic Class	*Well differentiated*	25%	20%	**0.09**	24.2%	21.7%	0.81	24.7%	22.4%	0.30	24.8%	19.2%	0.22
	*Moderate or poorly differentiated*	45.7%	65.7%		49.2%	56.5%		53.8%	44.8%		47.2%	65.4%	

Microvascular Invasion		13.1%	23.7%	0.12	14.1%	24%	0.21	20.4%	8.1%	**0.04**	15%	18.5%	0.65

AFP, alpha fetoprotein; dNLR, derived neutrophil lymphocyte ratio; LMR, lymphocyte monocyte ratio; NLR, neutrophil lymphocyte ratio; PLR, platelet lymphocyte ratio.

**Table 3:** Patient and tumor characteristics effect on 5-year overall and recurrence free survival.

		5 Year Overall Survival		5 Year Recurrence Free Survival	
		HR (CI)	P value	HR (CI)	P value
Age		0.99 (0.96-1.03)	0.71	0.96 (0.91-1.02)	0.14

Gender	*HR of Female to Male*	1.19 (0.58-2.43)	0.64	0.30 (0.04-2.35)	0.25

Race	*HR of other races to White, non-Hispanics*	1.36 (0.68-2.73)	0.38	3.56 (1.15-11.05)	**0.03**

Etiology	*HR HCV to non HCV*	1.21 (0.58-2.54)	0.61	0.69 (0.21-2.28)	0.54

MELD		1.01 (0.97-1.05)	0.73	1.00 (0.93-1.07)	0.99

AFP	*HR of >300 to ≤300*	3.16 (1.40-7.15)	**0.006**	8.78 (2.64-29.24)	**0.0004**

Tumor on explant		1.38 (0.58-3.29)	0.47	2.72 (0.35-21.07)	0.34

Histological Stage	*T1*	1.55 (0.44-5.43)	0.50	9236	0.93
	*T2*	2.00 (0.59-6.61)	0.27	28440	0.93
	*T3*	2.08 (0.35-12.44)	0.42	82832	0.92

Histological class	*Well differentiated*	0.67 (0.25-1.76)	0.41	0	0.97
	*Moderate or poorly differentiated*	1.00 (0.47-2.14)	0.99	5.02 (0.64-39.18)	0.12

Microvascular invasion		2.36 (1.20-4.64)	**0.01**	4.05 (1.28-12.75)	**0.02**

NLR	*HR of ≥5 to <5*	1.75 (0.91-3.35)	**0.09**	1.64 (0.50-5.46)	0.42

dNLR	*HR of ≥3 to <3*	1.76 (0.86-3.61)	0.12	1.87 (0.51-6.92)	0.35

LMR	*HR of ≥3.45 to <3.45*	0.62 (0.32-1.22)	0.17	0.29 (0.06-1.34)	0.11

PLR	*HR of ≥150 to <150*	3.18 (1.66-6.11)	**0.001**	7.95 (2.52-25.09)	**0.0004**

AFP, alpha fetoprotein; dNLR, derived neutrophil lymphocyte ratio; HCV, hepatitis C virus; HR, hazard ratio; LMR, lymphocyte monocyte ratio; MELD, Model for End Stage Liver Disease; NLR, neutrophil lymphocyte ratio; PLR, platelet lymphocyte ratio.

## Data Availability

The data used to support the findings of this study are available from the corresponding author upon request.
